# A case of angioimmunoblastic T-cell lymphoma presenting with migration of lung shadows

**DOI:** 10.1016/j.rmcr.2023.101972

**Published:** 2023-12-29

**Authors:** Tomoya Maruyama, Takashi Ishiguro, Kenji Takano, Yoshihiko Shimizu

**Affiliations:** a Department of Respiratory Medicine, Saitama Cardiovascular and Respiratory Center, Saitama, Japan; b Department of Pathology, Saitama Cardiovascular and Respiratory Center, Saitama, Japan

**Keywords:** Angioimmunoblastic T-cell lymphoma, Wandering, Epstein-Barr virus, Common variant immunodeficiency, Migration

## Abstract

A 62-year-old woman presented with chronic cough. Chest CT showed multiple nodules and consolidation. Bronchoscopy could not confirm a specific diagnosis. Because her symptoms and lung opacities improved spontaneously, she was followed without treatment. Seven months later, chest radiography showed worsening of consolidation and a tumorous shadow. After performing cervical lymph node and lung tissue biopsies, we diagnosed her as having angioimmunoblastic T-cell lymphoma (AITL). Cases of AITL showing migration of lung shadows have not been reported. AITL development is influenced by immunodeficiency and reactivation of EBV, and migration of lung opacities may be related to the patient's immune status.

## 1 Introduction

Pulmonary opacities, which repeatedly appear and disappear over the natural course of disease, are often explained as “wandering” or undergoing “migration” because they seem to move. Infectious or non-infectious pulmonary diseases show wandering lung shadows. Here, we report a case of angioimmunoblastic T-cell lymphoma (AITL) that showed wandering shadows. This would appear to be a rare case because, to our knowledge, no cases of AITL presenting with migrating shadows have been reported.

## 2 Case presentation

A 62-year-old Japanese woman presented to our hospital complaining of coughing for 2 months, and bilateral abnormalities were revealed on chest radiography. Her past medical history included subarachnoid hemorrhage at 50 years of age. She had not started taking any new medications for several years. She smoked 10 cigarettes a day for 27 years, stopping at 50 years old. She had no history of drinking or exposure to chemicals or dust and no family history of respiratory diseases or malignancy. On admission, her body temperature was 36.3 °C and SpO_2_ was 97 % on ambient air. Chest auscultation revealed normal sounds. There were no superficial swollen lymph nodes, skin rash, or pitting edema. Arterial blood gas analysis on ambient air showed pH 7.52, PaCO_2_ 34.8 Torr, PaO_2_ 66.6 Torr, and HCO3^-^ 28.8 mmol/L. Laboratory tests showed lymphocytopenia: her white blood cell count was 4800/μL (neutrophils 81.1 %, eosinophils 3.8 %, lymphocytes 6.9 %), hemoglobin 13.7 g/dL, platelets 31.1 × 10^4^/μL, and immunoglobulin levels were low: IgG 387 mg/dL, IgA 114 mg/dL, IgM 50 mg/dL, and IgE 17 IU/mL. Lactate dehydrogenase was 276 IU/L, C-reactive protein was 1.68 mg/dL, and soluble interleukin-2 receptor was 1430 U/mL (reference range: 121–613 U/mL). Aminotransferase and creatine were within normal range. Autoantibodies related to connective tissue diseases such as anti-nuclear antigen, rheumatoid factor, or anti-neutrophil cytoplasmic antibody were negative, tumor markers were not elevated, and serum cryptococcal antigen was negative. Urinalysis results were within normal range.

**Fig. 1:**
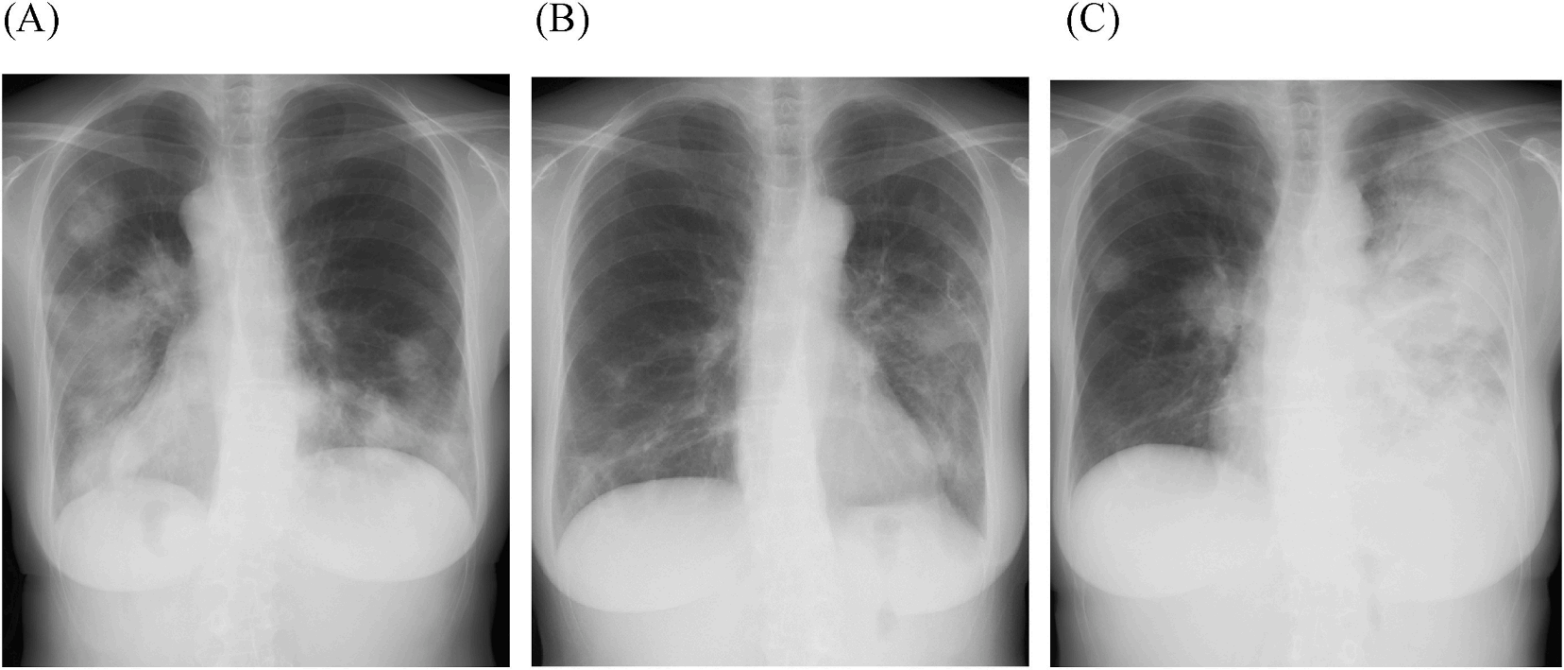
Chest X-rays of the patient. (A) Image at first visit to our hospital showed bilateral multiple tumorous and nodular shadows. (B) Image obtained 3 months after first visit showed improvement of opacities in the right lower lung field, and nodular shadows were partly enlarged in the left lung. (C) Image at 7 months after first visit showed extensive consolidation and enlargement of tumorous shadow in the left lung field. Nodular shadows were also present in the right hilum and the middle right lung field.

**Fig. 2:**
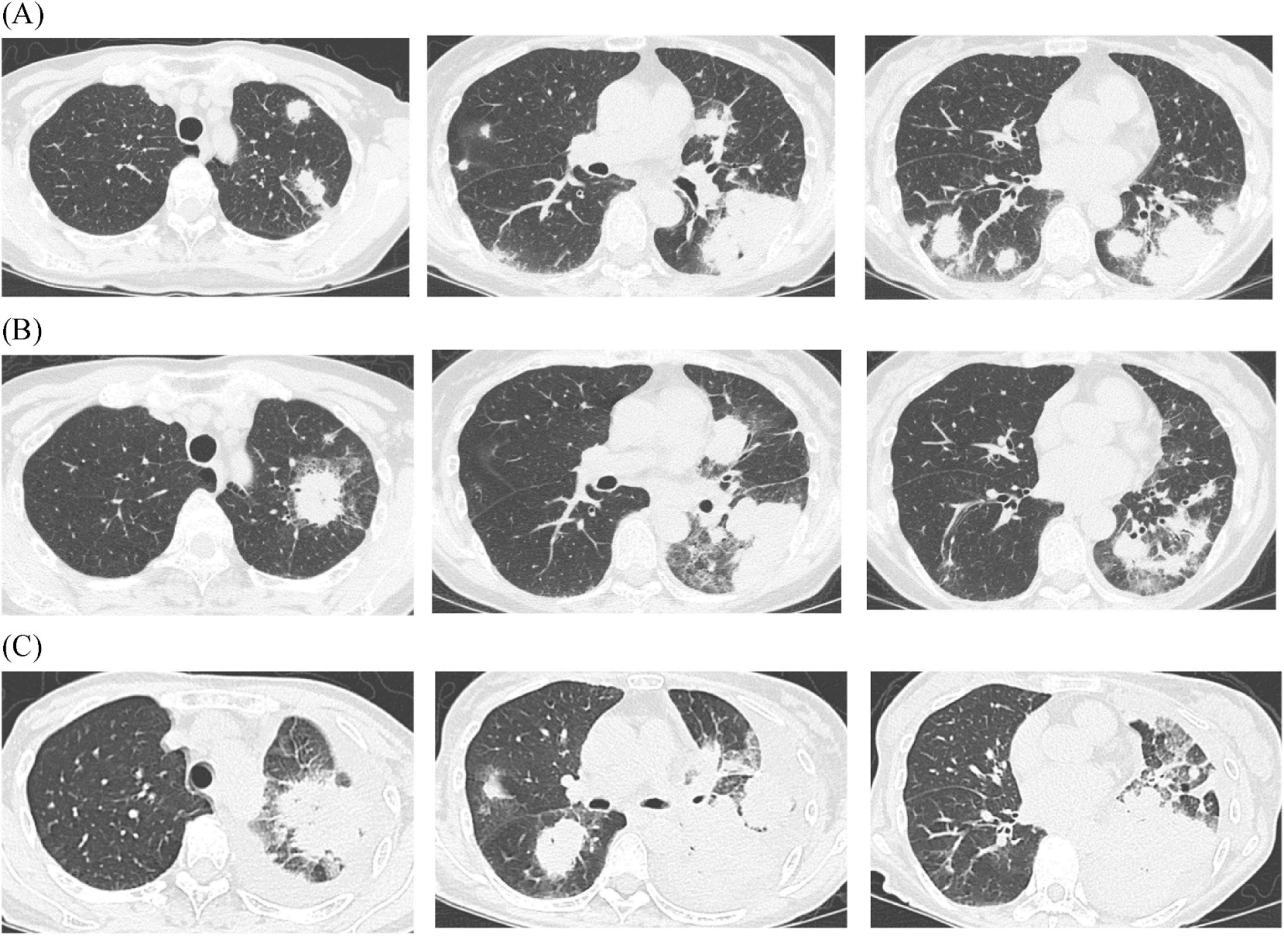
Chest CT images of the patient. (A) Image at first visit to our hospital showed multiple tumors and nodules in the bilateral lung fields. (B) Image at 4 months after first visit showed shrinking of tumors in the bilateral lower lobes and an enlarged nodule in the left lung. (C) Image at 7 months after first visit showed nodules were enlarged mainly in the left lung and a tumorous shadow emerged in the right lower lobe.

A chest radiograph showed consolidation and tumorous shadow mainly in the lower bilateral lung fields (Fig. 1A). There were shadows overlapping the cardiac outline and left pulmonary hilum, and the border of the lung field was indistinct. Chest computed tomography (CT) showed multiple nodules mainly in the bilateral lower lung fields, some of which were fused and formed tumorous shadow or consolidation (Fig. 2A). Ground-glass opacities and interlobular septal thickening were also present around nodules and tumor. CT of the paranasal sinuses showed slightly thickened mucosa of the maxillary sinus but no fluid accumulation.

The above findings indicated organizing pneumonia, granulomatosis with polyangiitis, cryptococcosis, and lymphoproliferative disorders as differential diagnoses. We performed bronchoscopy with bronchoalveolar lavage (BAL) in the left upper lobe (S3) and transbronchial lung biopsy in the left lower lobe (S9). We recovered 71 of 150 mL (47.3 %) of BAL fluid, which showed a white blood cell count of 4.0 × 10^4^/mL (neutrophils 0.2 %, lymphocytes 8.6 %, macrophages 91.2 %) and a CD4/CD8 ratio of 0.60. No atypical cells or hemosiderin-laden macrophages were present. No significant pathogens were isolated, and multiplex PCR of her BAL fluid was negative for viruses and *Mycoplasma pneumoniae* (FTD Resp 21 Kit; Fast Track Diagnostics, Silema, Malta). Transbronchial lung biopsy showed non-specific changes, so we could not confirm a diagnosis. Because her symptoms and lung opacities improved spontaneously after her discharge (Figs. 1B and 2B), and decreased lymphocytes and immunoglobulin were shown, we decided to observe her condition without administration of medication. The reduction in lymphocytes and immunoglobulin was examined in another hospital, and she was diagnosed as having common variable immunodeficiency (CVID).

**Fig. 3:**
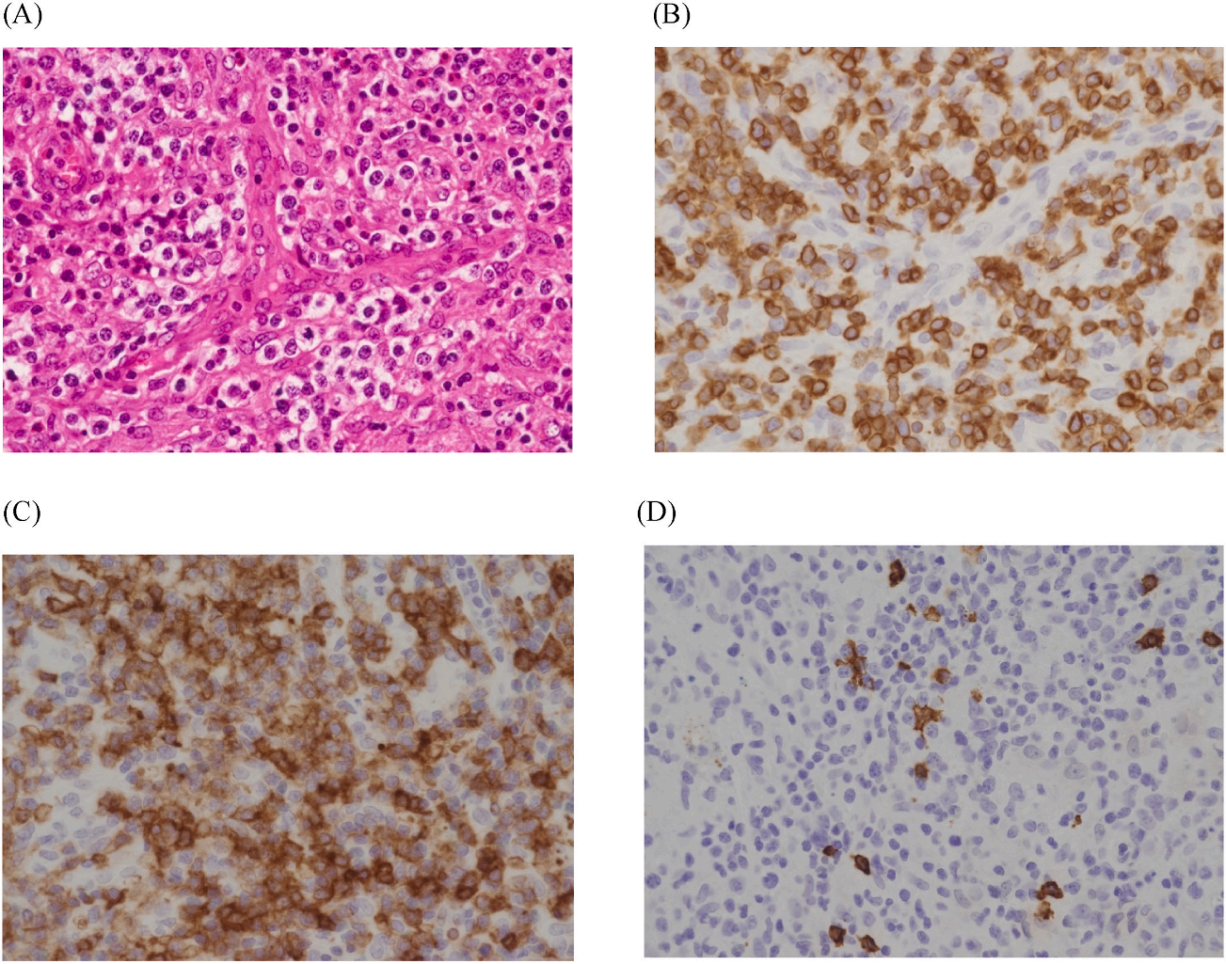
Pathological features of the right cervical lymph node. (A) HE staining revealed high endothelial venules (HEV) with thickened walls showing irregular dendritic branching and pale/clear cells increased around the HEV. Immunohistochemical staining revealed the atypical cells to be positive for CD3 (B), a marker of matured T cells, and CD10 (C), a marker of follicular helper T cells, and negative for CD20 (D), a marker of B cells.

**Fig. 4:**
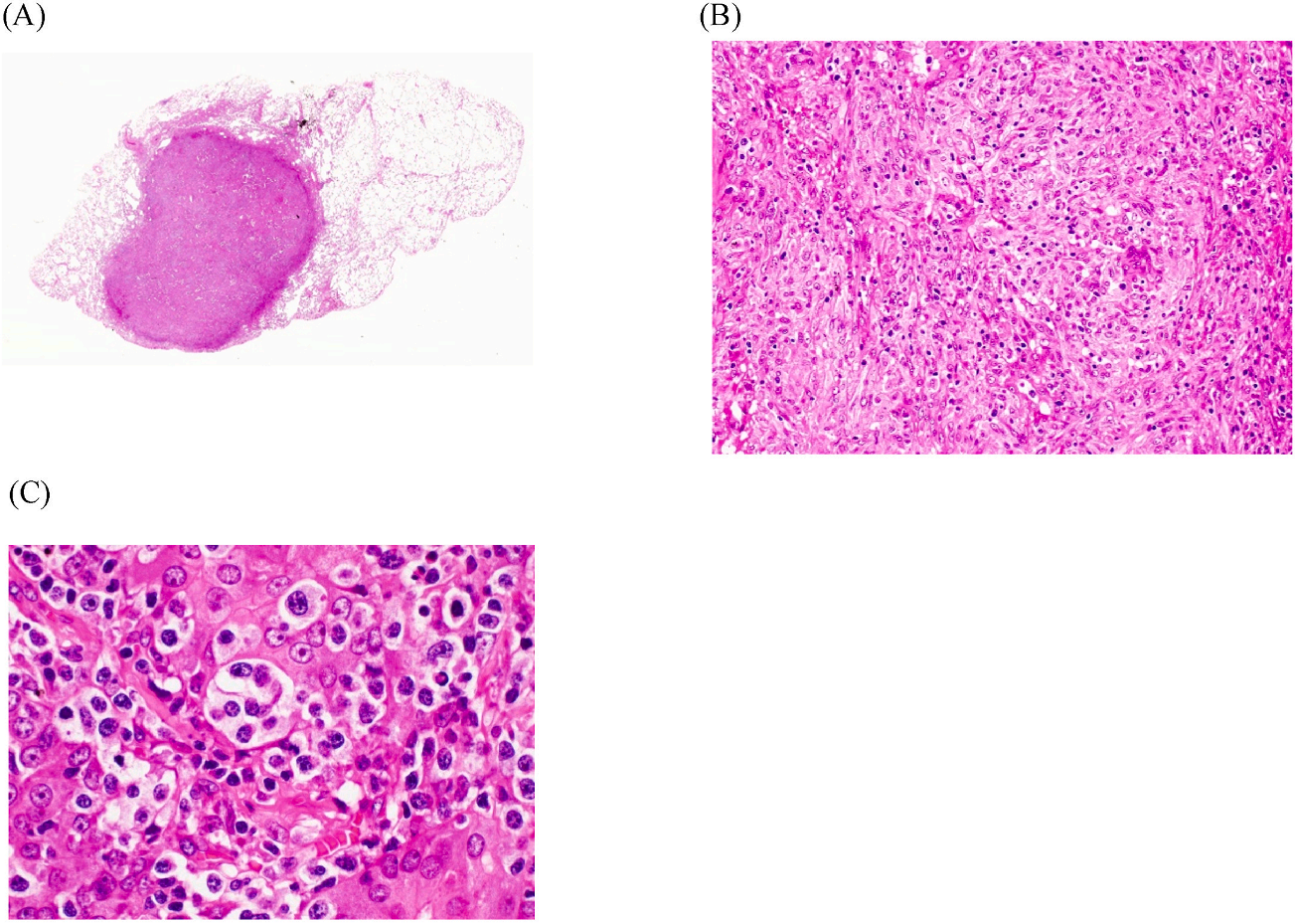
Pathological features of lung tissues from the surgical lung biopsy. (A) Panoramic image of HE staining showed a well-circumscribed nodular lesion. HE staining × 20 (B) and × 60 magnification (C) showed HEV and pale/clear cells similar to findings of the cervical lymph node.

Seven months after her first admission, she revisited us due to worsening of cough. A chest radiograph showed extensive consolidation and enlargement of a tumorous shadow in the left lung field (Fig. 1C). Nodular shadows had also emerged in the right hilum and middle right lung field. CT showed enlargement of nodules mainly in the left lung field (Fig. 2C) and multiple lymphadenopathies from neck to abdomen. A biopsy of her right cervical lymph node (Fig. 3) revealed high endothelial venules (HEV) with thickened walls showing irregular dendritic branching and increased pale/clear cells around the HEV. Immunohistochemical staining revealed atypical cells positive for CD3, CD5, CD10, bcl-2, PD-1, and ICOS, and negative for CD20 and CD79a. Additionally, follicular dendric cells positive for CD21 were increased around the HEV, and some of the B cells were positive for EBER-ISH. Her serum level of anti-EBV-VCA IgG was high and those of anti-EBV-VCA IgM and anti-EBV EBNA antigen were low, suggesting that she was already infected with EBV. We also performed surgical lung biopsy in the left upper lung, which showed similar findings to the biopsy of the right cervical lymph node (Fig. 4). From these histopathological manifestations, we diagnosed her as having angioimmunoblastic T-cell lymphoma (AITL). She received chemotherapy and autologous peripheral blood stem cell transplantation and has been followed up for several years.

## 3 Discussion

This is a case of AITL in which chest opacities repeatedly appeared and disappeared. We could not initially confirm the diagnosis, and chest radiographic findings showed spontaneous improvement. However, chest opacities emerged with worsening of symptoms, and we could finally diagnose AITL from histopathological manifestations of the cervical lymph node and lung biopsy.

AITL is defined as peripheral T-cell lymphoma characterized as a systemic lymph node-involved disease with diverse lymphocytic infiltrate and hyperendothelial venous and follicular dendritic cell proliferation [1]. AITL represents 1–2% of non-Hodgkin lymphoma and is more common in older adults, with the peak incidence in the sixties. Various symptoms are characteristic such as lymphadenopathy, hepatosplenomegaly, skin rash, fever, and weight loss. Although the prognosis of AITL depends on risk factors, it is reported to be poor with 5-year median survival of 32 % [2].

Low levels of lymphocytes and immunoglobulin were found in our patient, and she was initially diagnosed as having CVID. Malignant lymphoma sometimes develops in immunodeficiency patients. It is suggested that immunodeficiency causes infection or reactivation of pathogens associated with tumor development, such as EBV, and genetic variation in the embryonic cell lineage is related not only to primary immunodeficiency but also to development of lymphoma [3]. A case of AITL developing in a patient using immunosuppressive drugs was reported, which suggests the association of immunodeficiency with AITL [4]. Moreover, development of AITL is related to EBV infection [2]. In the present patient, reactivation of EBV due to immunodeficiency might have contributed to development of the AITL.

Lung involvement is reported in 7 % of AITL cases [5]. Chest imaging findings often include nodular shadows or consolidation, and enlarged mediastinal lymph nodes, pleural effusion, ground-glass opacities, and multiple nodules were also reported [[6], [7], [8]]. In the present case, besides formation of consolidation and a tumorous shadow, chest opacities spontaneously disappeared and reemerged. This pattern of chest imaging is labelled “migration”, and there are several diseases that show migration of shadows (Table 1). However, within our literature search of PubMed, we could find no cases of AITL that showed migration of shadows.

**Table 1:** Diseases that show migration shadow.

Infectious diseases	Mycoplasma pneumonia [11], Legionella pneumonia [12], Psittacosis [13], Virus pneumonia [14], Paragonimiasis
Non-infectious diseases	COP, CEP, Drug-induced lung disease, CTD-ILD, GPA, ABPM, Radiation pneumonitis [15], Crohn disease [16], Aspiration [17], PAP [18], Pulmonary infarction [18], Bronchioalveolar carcinoma [18], Pulmonary artery sarcoma [19]

COP = cryptogenic organizing pneumonia; CEP = chronic eosinophilic pneumonia; CTD-ILD = connective tissue disease-associated interstitial lung disease; GPA = , granulomatosis with polyangiitis; ABPM = allergic bronchopulmonary mycosis; PAP = pulmonary alveolar proteinosis.

Among lymphoproliferative diseases, migration of shadows was reported in a case of lymphomatoid granulomatosis (LYG) [9]. As mentioned above, development of AITL is associated with EBV infection, and LYG also develops by reactivation of EBV due to immunodeficiency. It was suggested that migration of shadows in LYG is associated with the balance between immune status and proliferation of EBV and tumor cells [10]. Cases of AITL with pulmonary involvement may show migration of shadows due to the same pathogenesis as with LYG.

## 4 Conclusion

We reported a case of AITL that showed migration of lung opacities. Migration of shadows in patients with AITL has never been reported; however, lung involvement of AITL should be considered as a differential diagnosis when migration of shadows is present on lung imaging.

## Declaration of competing interest

The authors declare that they have no known competing financial interests or personal relationships that could have appeared to influence the work reported in this paper.
